# Clinical Findings in SMR Neurofeedback Protocol Training in Women with Fibromyalgia Syndrome

**DOI:** 10.3390/brainsci11081069

**Published:** 2021-08-15

**Authors:** Carlos Barbosa-Torres, Sixto Cubo-Delgado

**Affiliations:** 1 Department of Psychology, University of Extremadura, 10003 Cáceres, Spain; 2 Department of Educational Sciences, University of Extremadura, 06006 Badajoz, Spain; sixto@unex.es

**Keywords:** fibromyalgia, central sensitization syndrome, neurofeedback, perceived pain, sensorimotor rhythm, treatment, EEG operant conditioning

## Abstract

Fibromyalgia is related to central sensitization syndrome (CSS) and is associated with chronic pain and a decrease in general health. The aim of this study was to explore how changes in brain patterns of female fibromyalgia patients are shaped by neurofeedback therapy and how it affects pain perception and general health. A quasi-experimental study with pre- and post-tests was carried out with 37 female fibromyalgia patients referred by the Pain Unit of the National Health Service of Spain. The method involved applying a sensorimotor rhythm (SMR) protocol to monitor changes in brain waves under different conditions, taking pre-/post-test measurements of perceived pain, general health and the impact on fibromyalgia. Measures included the Fibromyalgia Impact Questionnaire Revised (FIQR), the Visual Analogue Scale (VAS), the General Health Questionnaire (GHQ-28) and EEG (SMR, theta waves). During therapy, the SMR/theta wave ratio increased significantly and after application of therapy, significant results were observed for the FIQR, VAS and GHQ-28. In conclusion, neurofeedback therapy increases the SMR/theta wave ratio in fibromyalgia, helping to maintain a balance between brain functions. This is associated with the activation of inhibitory processes, which is related to the perceived improvement of pain in fibromyalgia patients.

## 1. Introduction

Fibromyalgia (FM) is a condition generally associated with multiple symptoms, such as sleep deprivation, tiredness, chronic fatigue and cognitive impairment [1,2]. Its predominant characteristic is non-articular, widespread, chronic muscular-skeletal pain in specific areas all over the body [3,4]. The psychological factors most frequently found in fibromyalgia patients are intense negative emotions (anxiety, depression), a maladaptive coping style, an unadjusted attention pattern and an excessive worry response [5,6].

The diagnosis of fibromyalgia most accepted by researchers focuses on the symptoms of central sensitization syndrome (CSS) [7]. The main supporting hypothesis is that pain is received, amplified and then permanently fixed by the brain. Current techniques are unable to disrupt the sensitization process in the brain regions where it occurs. This process is called suprasegmental central sensitization because it can take place across multiple regions, such as the thalamic area and the sensory cortex. Changes in inhibitory mechanisms and, especially, their disruption are considered an inherent part of discussions of the pathology of the condition [8,9,10]. Neuroimaging and EEG studies have suggested that hyperexcitability of the central nervous system represents an important mechanism in the maintenance of chronic pain in such patients [11,12]. Microstructural changes in some of these areas of neurochemical origin play an important role in the maintenance of pain as well as in its presence related to affective symptoms [13]. Fibromyalgia patients show greater brain connectivity in regions that are responsible for pain processing and simultaneously, they also have a significant reduction in connectivity in regions that are responsible for pain inhibitory modulation [14].

According to Donaldson, not all patients with fibromyalgia have the same rhythmic brain pattern. In general, patients demonstrate a stronger potential during slow waves, with deteriorations in both alpha rhythms (8–12 Hz) and sensorimotor rhythms (SMRs) (12–15 Hz). Some patients also show other rhythmic brain patterns [15,16].

Neurofeedback is a non-invasive technique that involves using operant conditioning in order to train how to control brain wave activity [17]. A specific form of biofeedback is used, in which electrophysiological processes are monitored by an electroencephalogram (EEG) [18,19] that allows direct intervention at the cortical level by reorganizing electrical signals to achieve central desensitization and provide relief from symptoms [20].

Sensorimotor rhythms are generated in the thalamus and range from 12 to 15 Hz [21,22]. The neuronal mechanism by which SMRs are formed is the interaction between the cerebral cortex and the thalamic ventrobasal complex relay nuclei, associated with somatosensory afferent suppression. When sensorimotor rhythms occur, the discharge pattern of the ventrobasal complex nuclei changes from rapid and non-rhythmic to rhythmic and systemic. This event is associated with suppression of somatosensory information and a reduction in muscle tone [23].

SMRs have been studied because of the capacity people develop for training and, subsequently, how they regulate SMRs voluntarily [24,25,26]. Therefore, training of sensorimotor rhythms could aid in reorganizing the intrinsic pathways found in pain perception amplification systems [27]. The amplitude of the P300 wave is lower in fibromyalgia patients compared with people without any pathology [28,29]. Therefore, the P300 wave is a reflection of the inhibition of the nervous system and if its amplitude increases with SMR training, then both are related to suppression of the transmission of painful information [30]. Studies on SMR-based neurofeedback training in chronic fibromyalgia patients have shown significant improvements in pain relief and other non-pain-associated symptoms [31,32].

Using a baseline comparison of controlled psychological test variables, a significant decrease was observed in the SMR/theta wave ratio at the end of treatment [33,34]. The aim of this study was to explain how the neurofeedback (EEG operant conditioning) intervention affects physiological variables, such as the amplitude of the SMR/theta wave ratio and how this is reflected in the pain perceived and the general well-being of female fibromyalgia patients.

## 2. Materials and Methods

### 2.1. Patients

The study was conducted with 37 patients referred from the Pain Unit of the Spanish National Health Service in Badajoz, Spain. All participants were women, with a mean age of 54.92 years (SD = 7.89). The inclusion criteria were age 18 years of age or more and a fibromyalgia diagnosis based on the criteria set by the American College of Rheumatology (ACR). The exclusion criteria were other health problems, such as coronary diseases, cancer, or neurological disorders, such as epilepsy and patients voluntarily receiving any other treatment while participating in the study.

### 2.2. Design and Procedure

This was a quasi-experimental study of a single group with pre- and post-tests. The neurofeedback training was administered three times a week for seven consecutive weeks. A total of 20 sessions were carried out which had a duration of 15 min each, adding 5 min for the placement of silver cup electrodes for each patient. All safety standards and standardized protocols for the application of this type of therapies were met [35]. A validated standardized sensorimotor rhythm (SMR) protocol was used for training, which consisted of increasing sensorimotor rhythms and decreasing theta waves. Before therapy, all participants completed a questionnaire related to pain, which was repeated at the end of training. The participants were informed of the feedback system and the purpose of the training was explained to them (increase SMR and decrease theta waves) with open instructions. They were instructed to pay attention to maximize their scores. At the beginning of the training, each patient was invited to sit comfortably in front of a computer screen in a stress-free room. The training consisted of a puzzle, which the participants had to complete and they were rewarded with puzzle pieces and auditory beeps through visual and auditory stimuli. In addition, band amplitude values were transformed into visual feedback representations.

All subjects gave their informed consent for inclusion before they participated in the study. The study was conducted in accordance with the Declaration of Helsinki and the protocol was approved by the Doctoral Program Committe of Extremadura University with the identification code (R014).

### 2.3. Outcome Measures

The Fibromyalgia Impact Questionnaire Revised (FIQR) consists of 21 items with a total score from 0 to 100 that measures the overall impact (scores from 0 to 20), physical dysfunction (scores from 0 to 30) and intensity of symptoms over the previous week (scores from 0 to 50). The total score is obtained by adding the subscales. Higher scores are associated with a greater impact, where patients score on average about 50 and severely affected patients score 70 or more. The Spanish version of the FIQR is internally consistent (α = 5; 0.91–0.95) with adequate test–retest reliability (r = 0.82) [36,37].

The Visual Analogue Scale (VAS) allows measuring the pain intensity described by the patient, with the maximum reproducibility among observers. Lower or absence of intensity is located on the left side, whereas higher intensity can be found on the right side. The patient marks the line to indicate the intensity, which is measured with a millimeter ruler and the intensity is expressed in centimeters or millimeters anchored by two descriptors, no pain and unbearable pain, with a total score from 0 to 10. The assessment is (1) mild pain if the patient scores the pain as less than 3, (2) moderate pain if the assessment is between 4 and 7 and (3) severe pain if the assessment is equal to or greater than 8 [38].

The General Health Questionnaire (GHQ-28) comprises 28 items and 4 subscales that present, items of somatic symptoms (weakness, illness and bodily discomfort), anxiety (tension, anxiety and sleep), social dysfunction (problems enjoying daily activities) and severe depression (thoughts and feelings of sadness, hopelessness and suicide). A score of 6 was considered the cut-off in this study, according to the response system «0,0,1,1» by Lobo (1986) in the Spanish version. The predictive validity under these conditions, used for the clinical population, presented a sensitivity of 84.6% and a specificity of 82% [39].

### 2.4. EEG Data Acquisition and Preprocessing

The instruments used for collecting and analyzing electrophysiological variables are designed by the Thought Technology Company. The ProComp Infiniti System Encoder (model T7500M) is a multi-channel device used for real-time psychophysiological analysis, neurofeedback training and data acquisition [40]. The main measurement was the amplitude of the SMR and theta waves produced during the training sessions. Measurements were taken using an EEG with a monopolar assembly in C4 placed over the sensorimotor cortex on the right side of the scalp (according to the standard 10–20 system), with the ground electrode on the right earlobe. A sampling rate of 250 Hz was used and band-filtered to extract theta waves (4–8 Hz) and the SMR (12–15 Hz) using BioGraph proprietary software. The impedance was set under 5 kΩ using an electrode impedance checker. Fiber-optic communication was used between the EEG device and laptop with slow channels of 3.6–6.5 V and 7.260 ± 1.969 V EEG sensor supply voltage.

The software used was the BioGraph Infiniti platform (version 6.0) [41], which captured and analyzed data. Information was collected through two different procedures. The first procedure corresponded to 20 sessions of neurofeedback for each patient (open display session). The second procedure was the pre-/post-test baseline (script session) obtained from each patient before and after treatment in order to retrieve data that could compare the brain state under four specific conditions: A1, eyes open baseline (without activity); A2, eyes closed baseline (without activity); A3, sensory attentiveness (active listening to a 4 min audio); and A4, cognitive effort (visual search for numbers on the screen). Under the first two conditions, the participants had to remain relaxed with their eyes open and closed, respectively. The pre-test measurement was carried out 1 day before treatment and the post-test measurement 1 day after treatment.

### 2.5. Statistical Analysis

Statistical data analysis was carried out using SPSS v.25 software. The Shapiro–Wilk test (*p* ≤ 257) applied to test the theoretical population was normal, in contrast to the null hypothesis. The runs test (*p* ≤ 137) was performed to check that the null hypothesis of the theoretical distribution in the population was random. In relation to these results, the necessary statistical model was applied to analyze the working hypothesis. The Wilcoxon signed-rank test was performed to compare related samples between the pre- and post-test of each type of wave and experimental condition and the Friedman test to compare the 20 sessions of training. Direct scores were used for FIQR, VAS and GHQ-28 analyses. When assessing the impact of an intervention, in addition, to its statistical significance (*p* ≤ 0.05), Cohen’s d was used to analyze the effect size.

## 3. Results

Table 1 shows the descriptive descriptors of the sample.

The amplitude (μV) of the SMR, theta waves and their ratio showed significant changes (SMR, *p* = 0.010; theta waves, 168 *p* < 0.001; ratio, *p* < 0.001) during the 20 sessions (open display session). In the analysis of the pre- and post-tests (script session), sensorimotor rhythms increased and theta waves decreased by the application of neurofeedback EEG. Regarding SMRs, under the first three conditions (A1, *p* < 0.001; A2, *p* = 0.007; A3, *p* = 0.026), there was a significant increase in amplitude, but under the last condition (A4, *p* = 0.272), no significant data were produced. All theta waves significantly decreased in amplitude (A1, *p* < 0.001; A2, *p* = 0.026; A3, *p* = 0.019; A4, *p* < 0.001). In addition, the SMR/theta wave ratio was increased by applying neurofeedback EEG; however, significant data were found under the first three conditions (A1, *p* < 0.001; A2, *p* = 0.002; A3, *p* < 0.001) but not under the last condition (A4, *p* = 0.312).

A statistically significant difference on the Fibromyalgia Impact Questionnaire Revised (FIQR) was observed, with a mean pre-test score of 76.7, a mean post-test score of 63.5 (*p* =< 0.001) and a large effect size (*d* = 0.940). In the Visual Analogue Scale (VAS), there was a statistically significant decrease in the mean pre- and post-test scores (8.4 and 6.3, respectively; *p* < 0.001), with a large effect size (*d* = 0.805). In the General Health Questionnaire (GHQ-28), the mean pre- and post-test scores were 17.4 and 13.5, respectively, and there was a significant reduction in the total score (*p* = 0.011), with a large effect size (*d* = 0.536). The only two dimensions that exceeded the cut-off in the pre-test were anxiety and social dysfunction. The post-test scores decreased significantly for both dimensions (*p* < 0.001), as well as for dimensions that did not pass the cut-off in the pre-test. The rest of the dimension scores are given in Table 2.

## 4. Discussion

In relation to brain waves, the conclusions are presented in two ways and are based on wave progression during the 20 sessions (open display session), the data obtained in the pre-/post-test baselines (script session) and the pre-/post-test analysis of the questionnaires. During the 20 sessions, the amplitude of the SMR significantly increased, while that of theta waves significantly decreased. As a result, a significant increase in the SMR/theta wave ratio was observed throughout the training.

Regarding the pre-/post-test baselines, evaluation of SMRs showed that the amplitude increased under conditions A1, A2 and A3 in contrast to the previous baseline, while it decreased under condition A4. Condition A4 might consume too many cognitive resources in fibromyalgia patients and prevent them from effectively concentrating during a task. Due to overexposure to sounds, numbers, shapes and colors could have a negative impact on results. Upon completion of condition A4, no testing was performed to measure whether the participants’ concentration levels were adversely affected by tiredness or whether they managed to maintain a level of alertness in accordance with the task requirements when looking at the screen. If the methods used to obtain pre-/post-test reference measures are reviewed, it would be necessary to modify the visual search activity (condition A4) due to the high level of challenge it presents, as well as to subjectively measure the participants’ concentration level. In the pre-/post-test baseline of theta waves, significant improvements were found under conditions A1, A3 and A4, while under condition A2, no significant results were obtained. It is estimated that with a larger sample group or a higher number of sessions, greater significance could be generated for all these measured electrophysiological variables. This is because the reorganization of brain waves is a complex process to understand and train, which is why patients display slow and not always ongoing changes. During this study, the participants adapted to the parameters set by the software used. Therefore, the required cognitive efforts made by the participants to influence their electroencephalograms were due to neurofeedback training. Changes in the location of the source of SMR and theta wave activities and the recording of metabolic activity during the increase or decrease in the theta waves through an fMRI should be analyzed and related to the main findings [31,42].

The data also showed that there was a significant improvement in pain perception and an impact on fibromyalgia symptoms. In a pilot study using the same training SMR protocol as ours, fibromyalgia patients showed improvements in sensitivity to pain and fatigue [43]. Another study corroborated that central nervous system hyperexcitability represents an important mechanism in the maintenance of chronic pain for such patients [42]. This could explain the reduction in perceived pain after an increase in the SMR/theta wave ratio in our study. Our results are in line with those of Sterman [44], who found that an increase in the SMR can facilitate the inhibitory mechanisms of the thalamus, which could be involved in the amplified perception of pain in fibromyalgia patients. It is important to note that the same brain structures responsible for the sensing and channeling of audio, visual, smell, taste and tactile stimuli also receive the sensation of pain. Influencing the inhibitory mechanisms of the central nervous system dampens the transmission of information of all sensory signals, especially pain, which is one of the key factors affecting the symptoms of fibromyalgia.

Our results are also consistent with those of Egner and Gruzelier [27], who explained that the SMR amplitude increases the magnitude of P300 waves, which facilitates thalamocortical inhibitory mechanisms. This finding could be one of the primary justifications for applying this type of training in fibromyalgia patients. Therefore, fibromyalgia patients who show brain autoregulation adequate to increase the amplitude of the SMR/theta wave ratio have perceived improvement of pain after training.

The discovery of electrical changes modifying brain physiology with training is an important finding. However, being able to give meaning to these processes and correlate them with certain types of behaviors and cognitive improvements is not easy, according to German neurologist Berguer [28]. Isolating certain electrical brain variables to reach a specific conclusion is difficult, since any change that occurs at the brain level affects multiple common processes.

In recent years, several studies have investigated neurofeedback in fibromyalgia patients [45]. Three of these studies were carried out with traditional neurofeedback with a sensorimotor rhythm protocol like the one used in our study. Results showed an improvement in chronic pain [34,43,46], in addition to the impact of fibromyalgia, anxiety, or depression, which is in line with our study. The other studies used various types of alternative neurofeedback (Flex neurotherapy, EEG-driven stimulation and low-energy neurofeedback) [47,48]. Review studies have highlighted the scarcity of data when comparing different publication results. Establishing correlations between them is a high-risk strategy, considering their use of different protocols or electrophysiological variables. The study variables under research define the objective of the investigation and, by implication, the results. If different procedures are compared, then they must be treated independently or at least related in the most scrupulously objective way possible. Furthermore, the effect of neurofeedback treatment should be compared with psychological or pharmacological rehabilitation in relation to the quality of life and chronic pain in fibromyalgia patients [49].

In future studies, it would be advisable to replicate the study under different conditions and designs (e.g., experimental group vs. control group; experimental group vs. experimental groups; compare cognitive requirement in different protocols or patients) to isolate the effect of participation.

Another variable that must be taken into account for future studies is the number of sessions and the effect that occurs during training. It would be advisable to obtain the optimal number of sessions so that the patient has significant learning without the appearance of the secondary effects of neurofeedback [50].

## 5. Conclusions

In this study, training was carried out on the somatosensory cortex and the neurofeedback increased the amplitude of the SMRs, indicating that the training induced neuronal plasticity [23] by increasing SMR activation during the task performance. Furthermore, changes in the EEG power and functional brain connectivity were reflected in a reduction in pain. Therefore, neurofeedback therapy with an SMR protocol is an adequate tool, with limitations, to improve pain chronification processes.

## Figures and Tables

**Table 1:** Characteristics of the patients.

Characteristics	(*n* = 37)	N (%)
Mean age, years (SD)	54.92 (7.89)	37
Pain symmetry	Yes	24 (64.8)
	No	13 (35.2)
Mean years since clinical diagnosis of FM	<5	11 (29.7)
	5–15	14 (37.8)
	15–25	10 (27.1)
	<25	2 (5.4)
Sick leave	Yes, at this moment	12 (32.4)
	No, at this moment	7 (18.9)
	Never	18 (48.7)
Pharmacological medication ^1^	Yes	35 (94.6)
	No	2 (5.4)
Physiotherapy treatment ^1^	Yes	19 (51.4)
	No	18 (48.6)
Psychological treatment ^1^	Yes	15 (40.5)
	No	22 (59.5)

^1^ Before neurofeedback training.

**Table 2:** Amplitude of waves during treatment sessions and pre- and post-test analyses.

Amplitude of Waves during Treatment Sessions				Pre- and Post-Test Analysis				
Sessions	SMR (µV)	Theta Waves (µV)	Ratio (µV)	Wave (Conditions)/Variables	Pre-Test Mean ± SD	Post-Test Mean ± SD	Wilcoxon Signed-Rank Test (*p*)	*d*
	Mean ± SD	Mean ± SD	Mean ± SD					
1	5.31 ± 4.94	14.86 ± 8.70	0.428 ± 0.079	SMR				
2	5.92 ± 3.89	12.97 ± 8.41	0.458 ± 0.094	A1	5.63 ± 3.84	7.72 ± 2.45	<0.001	0.773
3	4.77 ± 3.01	10.57 ± 6.48	0.503 ± 0.114	A2	6.32 ± 1.27	7.40 ± 2.59	0.007	0.529
4	4.60 ± 2.20	10.34 ± 4.31	0.469 ± 0.135	A3	6.85 ± 2.12	7.33 ± 2.38	0.026	0.213
5	5.29 ± 3.31	11.16 ± 8.80	0.478 ± 0.102	A4	6.58 ± 2.98	6.69 ± 1.49	0.272	0.047
6	4.96 ± 2.60	10.57 ± 5.99	0.482 ± 0.116	Theta wave				
7	5.74 ± 3.02	10.30 ± 6.83	0.513 ± 0.129	A1	14.29 ± 8.14	9.51 ± 9.34	<0.001	0.546
8	6.50 ± 3.28	11.52 ± 7.61	0.488 ± 0.106	A2	11.92 ± 8.47	9.78 ± 8.71	0.026	0.286
9	6.95 ± 3.22	12.47 ± 7.54	0.493 ± 0.111	A3	14.46 ± 7.02	10.96 ± 6.04	0.019	0.534
10	5.64 ± 2.24	10.26 ± 3.60	0.585 ± 0.208	A4	14.89 ± 7.76	12.69 ± 5.53	<0.001	0.327
11	6.04 ± 2.51	11.49 ± 5.45	0.484 ± 0.101	Ratio				
12	6.89 ± 4.82	12.15± 2.08	0.504 ± 0.114	A1	0.48 ± 0.19	0.63 ± 0.17	<0.001	0.832
13	6.06 ± 2.90	10.47 ± 4.86	0.488 ± 0.103	A2	0.53 ± 0.17	0.65 ± 0.15	0.002	0.749
14	5.96 ± 3.00	11.54 ± 6.77	0.473 ± 0.102	A3	0.43 ± 0.21	0.55 ± 0.16	<0.001	0.643
15	5.87 ± 2.82	9.82 ± 3.69	0.513 ± 0.117	A4	0.47 ± 0.18	0.45 ± 0.22	0.312	0.100
16	6.37 ± 2.76	10.04 ± 5.37	0.557 ± 0.146	FIQR	76.70 ± 17.41	63.5 ± 9.56	<0.001	0.940
17	5.62 ± 2.28	9.46 ± 4.93	0.586 ± 0.145	VAS	8.4 ± 2.4	6.3 ± 2.8	<0.001	0.805
18	6.12 ± 2.67	9.36 ± 5.10	0.603 ± 0.148	GHQ-28	17.4 ± 8.80	13.5 ± 5.35	0.011	0.536
19	6.56 ± 2.33	8.98 ± 4.15	0.648 ± 0.168	Somatic	3.21 ± 1.37	2.42 ± 1.05	0.002	0.659
20	6.46 ± 2.87	8.97 ± 4.64	0.633 ± 0.133	Anxiety	6.08 ± 1.85	4.42 ± 1.49	<0.001	0.988
**Friedman Test** (*p*)	0.010	<0.001	<0.001	Social	6.24 ± 1.04	5.36 ± 1.28	<0.001	0.755
				Depression	1.92 ± 0.98	1.31 ± 0.73	<0.001	0.706

Amplitude of waves during treatment sessions, SMR/theta wave ratio (1–20): Progression of brain waves throughout the intervention of neurofeedback. Pre- and post-test analysis under different conditions (A1, eyes open; A2, eyes closed; A3, sensory attentiveness; A4, cognitive effort) and variables (Fibromyalgia Impact Questionnaire Revised (FIQR), Visual Analogue Scale (VAS), General Health Questionnaire (GHQ-28)). PRE: pre-test; POST: post-test; *p* ≤ 0.05; Cohen’s *d*: effect size.

## Data Availability

Data are available from the corresponding author upon request.
